# Pornography Use Could Lead to Addiction and Was Associated With Reproductive Hormone Levels and Semen Quality: A Report From the MARHCS Study in China

**DOI:** 10.3389/fendo.2021.736384

**Published:** 2021-09-10

**Authors:** Zhihong Cui, Min Mo, Qing Chen, Xiaogang Wang, Huan Yang, Niya Zhou, Lei Sun, Jinyi Liu, Lin Ao, Jia Cao

**Affiliations:** ^1^College of Pharmaceutical Sciences and Chinese Medicine, Southwest University, Chongqing, China; ^2^Institute of Toxicology, College of Preventive Medicine, Army Military Medical University, Chongqing, China

**Keywords:** pornography, addiction, reproductive hormone, semen quality, college students

## Abstract

This study aimed to investigate the situations of pornography use among male college students of China, to explore the addiction possibility for pornography use, and to study the associations between pornography use and reproductive hormone levels and semen quality. Five hundred sixty-eight participants met the inclusion criteria and finished all of the questionnaires and hormone level and semen parameter examinations. A majority of participants (except one) had pornography use experience, 94.2% participants started pornography use before college, and 95.9% participants reported they had masturbation experience when using pornography. Early contact to pornography, frequent pornography use, high amount of time spending on pornography use, and frequent masturbation during pornography use were correlated with addiction trends. Earlier pornography use was found to be associated with lower serum prolactin (PRL), follicle-stimulating hormone (FSH), and progesterone (Prog), as well as lower sperm concentration and total sperm count. Higher frequency of pornography use was associated with lower serum estrogen (E_2_). In conclusion, pornography use was common among male college students in China. Early contact, high frequent use, and high frequency of masturbation during pornography use could lead to addiction trends and aberrant reproductive hormone levels and semen quality.

## Introduction

Sex demand is considered an embarrassing issue in many eastern countries, particularly in China in the past years. It is hard for adolescents to get sex information from parents or schools. At the same time, adolescents during the puberty process have high demands for sexual sensation and sex information physically and psychologically. With the quick development of the Internet, pornography has become easily accessible to all ages because of its affordability, accessibility, and anonymity (1). It was estimated that 42.7% of Internet visitors have visited pornography websites, and 25% of Internet visitors visit pornography websites everyday (2). Adolescents are important audience for pornography works (3). An investigation among 563 US college students reported that by age 17, an overwhelming majority of boys (93%) and girls (62%) have been exposed to pornography, and boys were more likely to be exposed at an earlier age and with higher frequency (4). Similarly, situations happened in other regions (5–7). A 6-year longitudinal study showed that among middle school students of Hong Kong, from grade 7 (average age = 12.7) to grade 12, pornography consumption increased significantly, and boys were more likely to contact pornography works (8, 9). However, the pornography exposure to adolescents, especially to college students in mainland of China were not clear.

Some of the investigations showed that pornography had some positive or neutral effects on adolescents’ sexual practice. For example, pornography was found to be a resource to provide information about the human body, to increase the sense of sexual competence, and to decrease the sexual shame (10). On the other hand, there are also studies suggesting its negative effects. Several publications showed that increased pornography exposure was associated with earlier and quicker onset of sexual activity (11), more permissive attitude to casual sex (12), worse mental health (5), higher likelihood to risky sexual behaviors, and more acceptance of sexual violence (13). Moreover, a recent study revealed that problematic pornography user displayed a similar neural response as the drug addicts displayed (14). A study among Canadian university students (mean age 21) showed that daily and greater pornography use was associated with a sharp rise in addictive score (15).

Pornography is a specific sexual stimulus that can cause sexually related physical and psychological reactions, including sexual imagination, sexual erection, and masturbation (16). All of these behaviors were modulated by sexual hormones and feedback to hormone secretions, which was a typical feedback loop of hypothathalamus-putuitary-gonadal axis (HPG) activity (17, 18). Adolescents, including college students, are in the late stage of puberty, which is the important process of sexual hormones and organ maturation (19). However, none of the existing studies about pornography exhibited the effects of pornography on reproductive hormones and reproductive potentials.

The purposes of this study were as follows: (1) to explicate the current situation of pornography use among Chinese college students; (2) to investigate college students’ addiction trends for pornography use; and (3) to research the associations between pornography use and reproductive hormones and semen parameters.

## Materials and Methods

### Participants and Procedures

All of the participants in this study originated from a cohort termed the male reproductive health in Chongqing College students (MARHCS), which began in 2013 (20). This was the second follow-up study of the cohort. Since April 2015, all volunteers who participated in the baseline survey in 2013 received our cell phone and email illustrating this study, and they scheduled an investigation time from May 23, 2015 to June 7, 2015. All volunteers were asked to complete a questionnaire and underwent a physical examination. Individuals were included if they met the following criteria: over 18 years of age; 2–7 days of abstinence; no history of inflammation of the urogenital system, epididymitis, or testicular injury; no history of incomplete orchiocatabasis; and no history of varicocele treatment. Subjects were excluded if any of the following symptoms were detected by aurologist at the physical examination stage of the investigation: an absence of prominentialaryngea, pubis, or testis; abnormal penis or breasts; varicocele; or an epididymal knob. Five hundred sixty-eight males completed the survey, and all of them had a mean coming-out age of 22.4 years (SD = 1.2). Semen samples were collected and analyzed according to WHO guideline (WHO, 2010). Abstinence duration and ejaculation time were recorded. Peripheral blood was collected under aseptic conditions. The serum was isolated by centrifugation for serum reproductive hormone measurements. The Ethics Council of the Army Medical University approved the study, and informed consent was obtained from all subjects.

### Questionnaires

The questionnaires contained three parts: demographic information, lifestyle factors, and pornography use situations. The demographic information consisted of age, body mass index (BMI), and abstinence duration (days). Lifestyle factors included smoking, drinking, coffee consumption, cola consumption, and fried food consumption, which have all been reported to have an influence on semen quality (20).

The questions about pornography use situations and addictive behaviors were set according to previous studies about pornography exposure (21–25). Before the participants filled out the questionnaires, they were asked to read the definition of pornography, which included the following terms: sexually explicit material, sexually explicit media, pornography, porn, cyber-porn, Internet or online pornography, online erotica or erotica, and cyberpornography (24, 25).

### Serum Collection and Reproductive Hormones Detection

All collected serum samples were taken from a low-temperature refrigerator at −80°C and then sent to the Army Medical University Affiliated Southwest Hospital Laboratory. The serum samples were then liquefied and tested of six reproductive hormone levels, including estradiol (E_2_), follicle-stimulating hormone (FSH), luteinizing hormone (LH), progesterone (Prog), prolactin (PRL), and testosterone (T). The chemiluminescence method was used to determine the serum hormone levels, and the instrument was selected by Beckman’s fully automated immunoassay analyzer DXI 800 (Beckman Coulter Inc., Brea, CA, USA).

### Semen Collection and Analysis

Semen samples were collected by masturbation into a sterile, wide-mouth plastic container in an independent clean room. Then, the samples were incubated at 37°C for liquidation and were analyzed within 60 min. Semen volumes were measured by weighing, assuming that 1 ml of volume equals 1 g of weight. The semen parameters (motility, progressive motility, concentration, and total sperm count) were assessed with a computer-aided sperm analysis system (SCA CASA System; Microptic S.L., Barcelona, Spain) by a well-trained laboratory technician. Sperm morphology was identified by sperm smears using a Diff-Quick staining kit (Boruide, BRED-015). All semen analyses were performed according to the WHO criteria recommendations (26).

### Statistical Analysis

Semen parameters and sex hormones were presented as medians and percentiles. The Jonckheere-Terpstratest and Mann-Whitney *U* test were used to compare differences between the semen parameters of the groups. A multivariable-linear regression model was applied to exclude potential confounders (e.g., age, BMI, abstinence duration, tobacco smoking, alcohol drinking, coffee consumption, cola consumption, and fried food consumption), which have been reported to have effects on semen parameters (20). All semen parameters were log-transformed when they were nonnormally distributed. Additionally, all of the statistical data were analysis with the Statistical Package for the Social Sciences (SPSS, Chicago, IL, USA), and differences were considered statistically significant if *p* < 0.05, and all the tests were performed by two tailed.

## Results

### Demographic Characteristics of Participants, Lifestyles, Sex Hormones, and Semen Parameters

Descriptive results are demonstrated in **Table 1**. Five hundred sixty-eight participants met the inclusion criteria and finished the examination. The average age of the respondents was 22.4 years, and the average BMI value and abstinence duration were 22.2 (kg/m2) and 4.1 days, respectively.

**Table 1:** Demographic characteristics, lifestyle factors, reproductive hormone levels, and semen parameters of the participants.

Characteristics	Values (*n* = 568)
Demographic characteristics	
Age (years)^a^	22.4 ± 1.2
BMI (kg/m^2^)^a^	22.2 ± 2.9
Abstinence duration (days)^a^	4.1 ± 1.5
Lifestyle factors	
Tobacco smoking^b^	
Never	414 (72.9)
Quit	26 (4.6)
Current	128 (22.5)
Alcohol drinking^b^	
Never	122 (21.5)
Quit	22 (3.9)
Current	423 (74.5)
Reproductive hormones	
E_2_ (pg/ml)	30.6 ± 16.7
FSH (mIU/ml)	3.5 ± 1.7
LH (mIU/ml)	4.3 ± 1.7
PRL (ng/ml)	10.4 ± 5.0
Prog (ng/ml)	0.6 ± 0.4
T (ng/ml)	3.8 ± 1.1
Semen parameters	
Volume (ml)^a^	3.8 ± 1.9
Sperm concentration (×10^6^/ml)^a^	56 ± 45
Total sperm count (×10^6^)^a^	203 ± 183
Total motility (%)^a^	79 ± 16
Progressive motility (%)^a^	55 ± 17
Morphological normal spermatozoa (%)^a^	11.73 ± 7.15

a Values are presented as the mean ± SD.

b Value are presented no. (%).

The average E_2_, FSH, LH, PRL, Prog, and T concentrations were 30.6 pg/ml, 3.5 mIU/ml, 4.3 mIU/ml, 10.4 ng/ml, 0.6 ng/ml, and 3.8 ng/ml respectively. The average semen volume, sperm concentration, total sperm count, total motility, progressive motility, and morphologically normal spermatozoa count were 3.8 ml, 56 million/ml, 203 million, 79%, 55%, and 11.73%, respectively.

### Situations of Pornography Use

As **Table 2** shows, almost all of the male students (except one) had experienced pornography use, albeit to varying extents. A total of 84.2% of the students first searched for pornography during middle or high school, and approximately 45.3% of the students used pornography more than once per week. Additionally, approximately 51.6% of the students spent approximately 15 to 30 min using pornography at each use. Only 4.1% students reported they had never masturbated during pornography use, when asked about their masturbation frequency when using pornography. In contrast, 14.9% of the students masturbated almost every time they used pornography.

**Table 2:** The distribution of pornography use-related questions.

Characteristics	*N*	Percentage (%)
**Situations for pornography use**		
Q1: First time to contact with pornography information (*n* = 568)		
Primary school	57	10.0
Middle school	277	48.8
High school	201	35.4
College	32	5.8
Never	1	0.18
Q2: Pornography use frequency (*n* = 567)		
<1 time/week	310	54.6
1–2 times/week	210	37.0
>2 times/week	47	8.3
Q3: Amount of time spending on pornography use/time (*n* = 567)		
≤15 min	201	35.4
15–30 min	293	51.6
≥30 min	64	12.9
Q4: Frequency of masturbation when using pornography (*n* = 567)		
Never	23	4.1
Sometimes	295	52.0
Half of the time	129	22.8
Most of the time	92	16.2
Almost every time	28	14.9
**The addiction possibility for pornography use**		
Q5: Frequency of pornography use in the most recent 3 months compared with 3 months ago (*n* = 567)		
Much less than before	162	38.6
A little bit less than before	126	22.2
As much as before	243	42.9
More than before	36	6.4
Q6: Is it take more time to feel sexual excitement when using pornography compared with 3 months ago (*n* = 567)		
Yes	180	31.7
No	387	68.3
Q7: Is it easier to achieve sexual satisfaction when using pornography compared with having sex with a real partner (*n* = 567)		
Yes	37	6.5
No	292	51.5
No sex partner	238	42.0

### The Possibility of Addiction on Pornography Use

Approximately half of the students reported they had used less pornography in the most recent 3 months. Additionally, 6.4% of the students reported they had used pornography more than before. One hundred and eighty (31.7%) students reported that they needed more time to feel sexual excitement than ever when using pornography. Excluding students who had no sexual partners (*N* = 238, 42%), 6.5% (*N* = 37) of the students reported that it was easier for them to achieve sexual satisfaction by using pornography than with a real sexual partner. Moreover, pornography use was significantly associated with addiction possibility. Earlier contact, more frequent use, longer time, and more masturbation during pornography use were all found to be correlated with addictive possibility (**Table 3**).

**Table 3:** Associations between pornography use and the addiction possibility for pornography use.

Addiction	First time to contact with pornography		Pornography use frequency		Amount of time spending on pornography use		Frequency of masturbation when using pornography	
	Correlation coefficient	*p*-Value	Correlation coefficient	*p*-Value	Correlation coefficient	*p*-Value	Correlation coefficient	*p*-Value
Frequency of pornography use in the most recent 3 months compared with 3 months ago	0.025	0.553	**0.260**	**<0.001**	0.035	0.405	**0.160**	**<0.001**
Is it take more time to feel sexual excitement when using pornography compared with 3 months ago	−0.007	0.866	−0.053	0.206	−**0.100**	**0.017**	0.005	0.915
Is it easier to achieve sexual satisfaction when using pornography compared with having sex with a real partner	**0.141**	**0.001**	0.075	0.076	**0.120**	**0.004**	**0.109**	**0.009**

The results in bold indicate that the variable was significantly associated with changes in semen parameters (p < 0.05).

### Correlations Between Pornography Use and Sex Hormones

**Figure 1:**
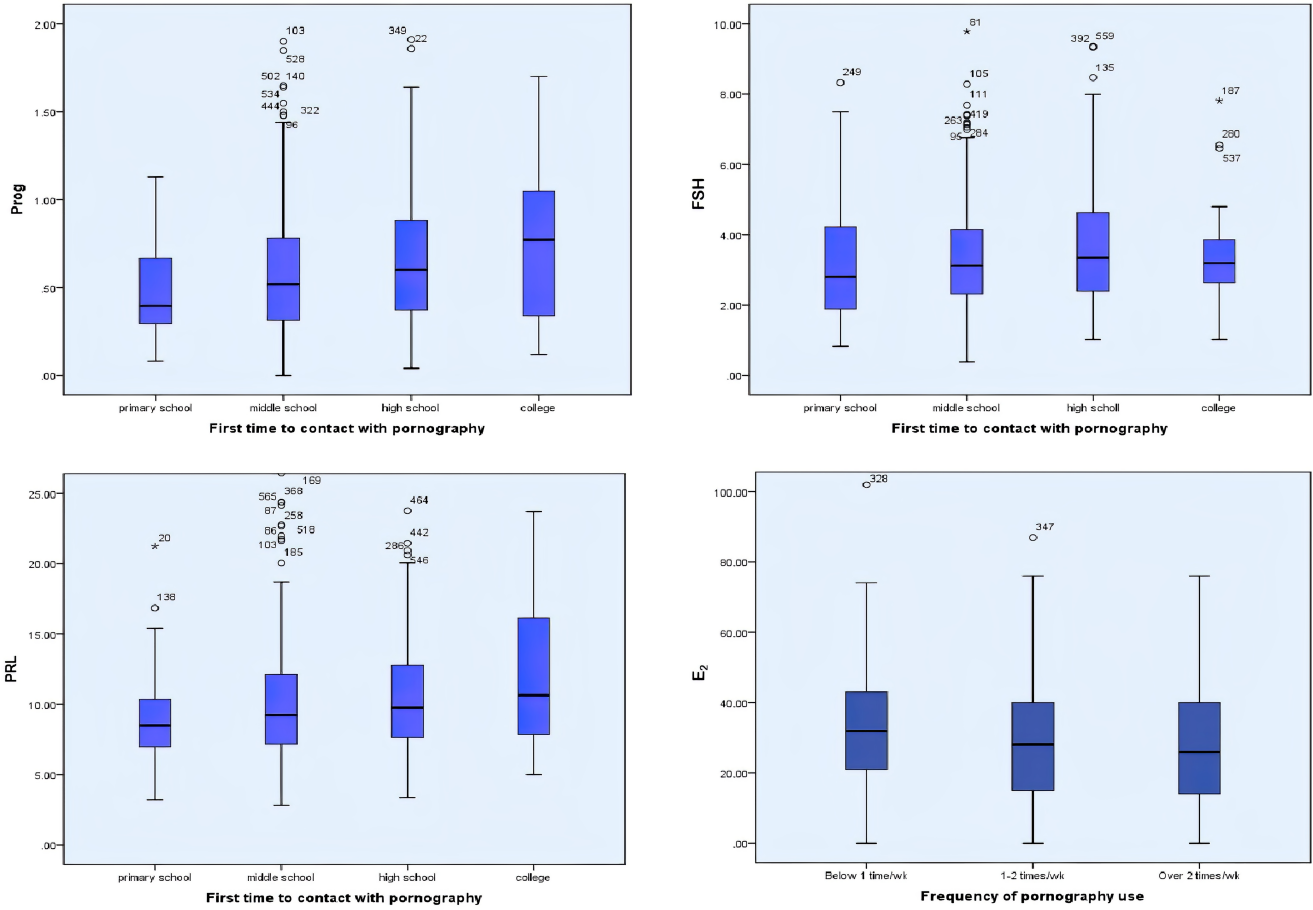
Univariate analyses were applied to estimate the associations between pornography use and sex hormones. The ages of first-time contact pornography were positively correlated with Prog, FS, and PRL concentrations in serum (*p* < 0.05, *p* < 0.05, *p* < 0.05). The frequency of pornography use were negatively correlated with E_2_ concentrations in serum (*p* < 0.05). *:extreme values.

Univariate analyses were applied to estimate the associations between pornography use and sex hormones. According to the increase of ages of “first time to contact pornography” from primary school to college, the Prog, FSH, and PRL concentrations were increased from 0.4 to 0.8 ng/ml, 2.7 to 3.2 mIU/ml, and 8.5 to 10.7 ng/ml, respectively (*p* < 0.05, *p* < 0.05, *p* < 0.05). The E_2_ concentration decreased from 32 to 26 pg/ml according to the increase of frequency of pornography use (*p* < 0.05) (**Figure 1**).

After adjusting the potential confounders (age, BMI, abstinence duration, tobacco smoking, alcohol consumption, coffee consumption, cola consumption, and fried foods consumption), students with higher pornography use frequency had lower E_2_ concentration in serum (*β* coefficient = −3.29; 95% confidence interval (CI), −5.14, −1.13, *p* = 0.003). Students with earlier exposure to pornography had lower PRL and Prog concentration in serum (*β* coefficient = 0.92; 95% CI, 0.37, 1.47, *p* = 0.001; *β* coefficient = 0.10; 95% CI, 0.05, 0.14, *p* < 0.001) (**Table 4**).

**Table 4:** Associations between pornography use and serum gonadal hormone levels.

Characteristics	E_2_		PRL		Prog	
	*β* (95% CI)	*p*-Value	*β* (95% CI)	*p*-Value	*β* (95% CI)	*p*-Value
First time to contact with pornography	0.06 (−1.82, 1.94)	0.953	**0.92 (0.37, 1.47)**	**0.001**	**0.10 (0.05, 0.14)**	**0.000**
Pornography use frequency	−**3.29 (**−**5.14,** −**1.13)**	**0.003**	−0.36 (−1.01, 0.29)	0.274	−0.02 (−0.08, 0.03)	0.378
Amount of time spending on pornography use	2.12 (−0.02, 4.25)	0.052	0.33 (−0.31, 0.97)	0.318	0.02 (−0.03, 0.08)	0.399
Frequency of masturbation when using pornography	−0.99 (−2.45, 0.48)	0.185	−0.16 (−0.60, 0.27)	0.466	−0.02 (−0.05, 0.02)	0.364

The results in bold indicate that the variable was significantly associated with changes in semen parameters (p < 0.05). Regression coefficients were adjusted for age, abstinence duration, BMI, smoking, and alcohol drinking status. A multiple linear regression analysis was deployed. The results are presented as regression coefficients with 95% confidence intervals.

### Correlations Between Pornography Use and Semen Parameters

**Figure 2:**
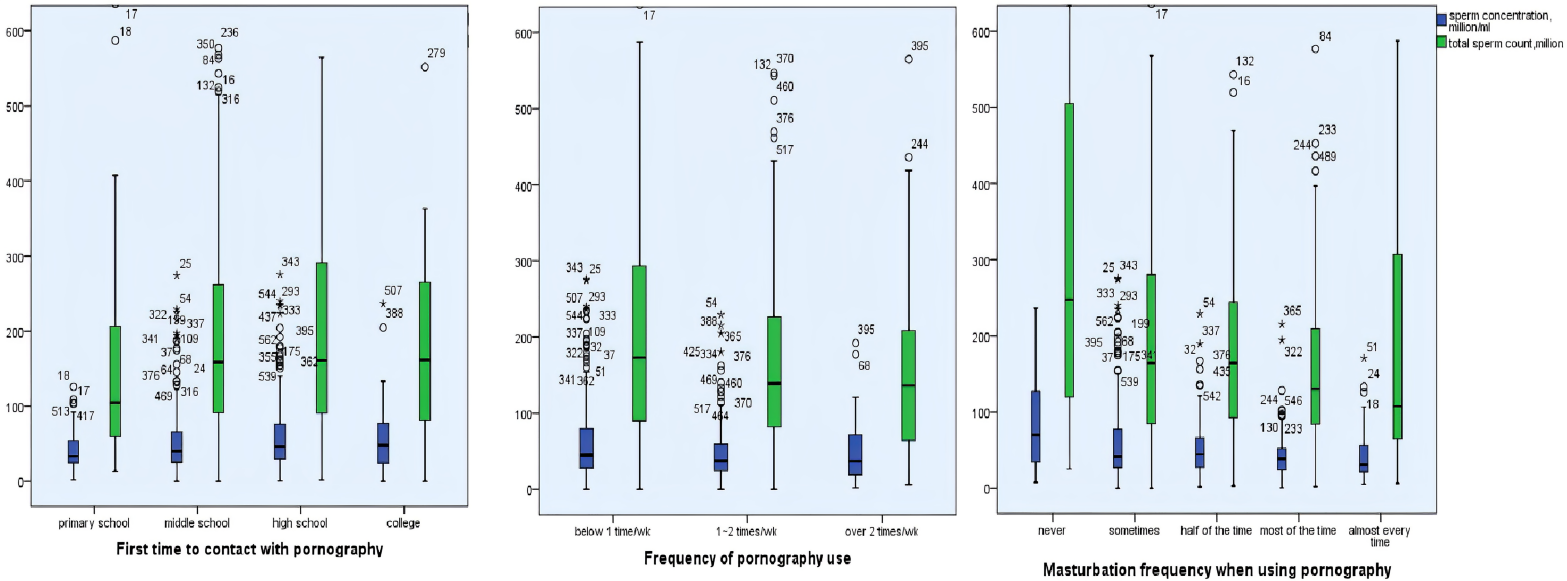
Univariate analyses were applied to estimate the associations between pornography use and semen parameters. The ages of first-time contact pornography were positively correlated with sperm concentration and total sperm count (*p* < 0.05). The frequency of pornography use and masturbation frequency when using pornography were negatively correlated with sperm concentration and total sperm count (*p* < 0.05, *p* < 0.05). *:extreme values.

Univariate analyses were applied to estimate the associations between pornography use and semen parameters. As shown in **Figure 2**, earlier pornography use, higher-frequency exposure to pornography, higher-frequency of masturbation when using pornography were associated with lower sperm concentration and total sperm count (*p* < 0.05).

Multivariable linear regression models were used to adjust the potential confounders (age, BMI, abstinence duration, tobacco smoking, alcohol consumption, coffee consumption, cola consumption, and fried foods consumption). After adjusting for potential confounders, the associations between pornography use and sperm concentration were still solid. The sperm concentration was positively associated with the ages of first time to contact with pornography (*β* coefficient = 11.17; 95% CI, 1.86, 21.34; *p* = 0.017) but negatively associated with masturbation frequency when using pornography (*β* = −7.40; 95% CI, −14.82, −0.46; *p* = 0.038). However, higher morphologically normal spermatozoa ratio (%) was found to be positively associated with masturbation frequency when using pornography (*β* = 7.40; 95% CI, 2.33, 12.72; *p* = 0.003) (**Table 5**).

**Table 5:** Associations between pornography use and semen parameters.

Characteristics	Sperm concentration (10^6^/ml)		Total sperm count (10^6^)		Morphological normal spermatozoa (%)	
	*β* (95% CI)	*p*-Value	*β* (95% CI)	*p*-Value	*β* (95% CI)	*p*-Value
First time to contact with pornography	**11.17 (1.86, 21.34)**	**0.017**	13.00 (−6.17, 32.17)	0.183	0.23 (−6.17, 6.91)	0.093
Pornography use frequency	−6.17 (−17.49, 3.99)	0.231	−19.09 (−42.00, 3.81)	0.102	0.00 (−7.89, 7.64)	0.992
Amount of time spending on pornography use	4.23 (−10.15, 19.40)	0.561	0.16 (−21.89, 22.20)	0.989	0.462 (7.89, 6.91)	0.883
Frequency of masturbation when using pornography	**−7.40 (−14.82, −0.46)**	**0.038**	−12.34 (−27.50, 2.82)	0.110	**7.40 (2.33, 12.72)**	**0.003**

The results in bold indicate that the variable was significantly associated with changes in semen parameters (p < 0.05). Regression coefficients were adjusted for age, abstinence duration, BMI, smoking, and alcohol drinking status. A multiple linear regression analysis was deployed, and all of the semen parameters were Log 10-transformed. The results are presented as back-transformed regression coefficients with 95% confidence intervals.

## Discussion

Adolescent pornography use is a hot public health issue (27, 28). The present study is an exploratory study designed to describe pornography use among college students in China and to explore the effects of pornography use on students’ reproduction health. We found that pornography use was quite common among college students in China. Pornography use was correlated with masturbation behavior and exposure to prolonged pornography may lead to addictive potential. Pornography use was also found to be significantly associated with sex hormone levels in serum and semen parameters.

Like in other countries or areas, pornography use among college students was quite common in China. In the present study, 94.2% of college students had contacted to pornography before college, which is consistent with other recent studies in other countries (29, 30).

The American Society of Addiction Medicine (ASAM) defined “addiction” in 2011 as: Addiction is a primary, chronic disease of brain reward, motivation, memory and related circuitry. Dysfunction in these circuits leads to characteristic biological, psychological, social, and spiritual manifestation. This is reflected in an individual pathologically pursuing reward and/or relief by substance and other behavior (31). Regarding pornography addiction, the clinical diagnostic criteria is still devoid. A number of studies supported the “self-reported pornography addiction” questionnaire to access the cognitive, behavioral, and emotional aspects of problematic pornography use (21–25). The questions about pornography use situations and addictive behaviors were set according to these previous studies. Quite a lot of studies led to the conclusion that frequent pornography use fitted into the addiction framework and shared a similar basic mechanism with substance addiction. The present study confirmed that pornography use might lead to addictive possibility; 31.7% of students reported they need more time to feel sexual excitement when using pornography, and nearly 6.5% of students thought it would be easier to achieve sexual satisfaction when using pornography than with a real sexual partner. Pornography has been reported to be a dopamine-producing base behavior (32). Dopamine is a neurotransmitter that is associated with activation of the brain’s reward system, and its presence helps initiate feelings of enjoyment and pleasure. Moreover, a study from the Max Planck Institute for Human Development showed that frequent pornography consumption was associated with the frontostriatal network, and pornography consumption hours were negatively associated with striatum volume. Individuals with a lower striatum volume may need more external stimulation to experience pleasure and might therefore experience pornography consumption as more rewarding (33). Therefore, frequent pornography use might break the balance of the dopamine pathway. Like heroin or other drugs, frequent pornography use may cause addiction, which might be a reason for why the pornography users showed difficulty in obtaining sexual excitement.

In the present study, we found that early pornography exposure was associated with lower adult FSH, Prog, and PRL levels in serum. After adjusting with potential confounders, we still found a significant association between “the first time contact with pornography” and Prog levels in serum. During puberty, the HPG axis is initiated, and the gonadal steroid hormones are dramatically increased (18), as well as the quick development of reproductive organs. FSH is responsible for starting the process of the sperm production (spermatogenesis) by initiating spermatogenic epithelium cell division and maturation. FSH have been also reported to be related with gonadotropin levels which lead to the induction of spermatogenesis effectively (34, 35). Recent studies showed that Prog produced by cumulus cells has been associated with various physiological processes in sperm production, including stimulation of acrosome reaction. Male normal prolactin levels help maintain a high level of testosterone in the testis and affect the growth and secretion of the gonadal glands (36). Hyperprolactinemia is a high level of serum prolactin, which can interfere with the periodic release of GnRH, the elimination of gonadotropin pulsed secretion, so that the release of LH and FSH decreased, eventually leading to hypogonadism (testosterone synthesis and secretion, spermatogenic dysfunction) (37). Thus, pornography use at an early stage may affect the function of the HPG axis and subsequently affect the secretion of steroid hormones, such as estrogen, androgen, prolactin, and progesterone, eventually affecting semen quality.

We also found that earlier pornography use was associated with lower sperm concentrations with or without adjusting to potential confounders. A majority of students are exposed to pornography before college. As we discussed in the previous section, adolescence is a key time for reproductive maturation, from sexual hormone secretion to sexual organ development. The time of puberty initiation and the time for first sexual encounter were found to be two developmental milestones associated with reproductive maturation (38). Earlier contact to pornography might lead to disturbance of sexual hormone secretion, and therefor lead to lower sperm concentration in adult semen. Besides, earlier contact to pornography might lead to higher frequency of masturbation, which is also a risk factor for lower sperm concentration. There are no other studies focused on male puberty pornography use and adult semen qualities, but some studies revealed that adolescents who were exposed to pornography were more likely to reach early sexual maturation and be higher sensation seekers (39).

The present study showed that pornography use was significantly correlated with masturbation behavior. Higher masturbation frequency during pornography use showed adverse effects on the sperm concentration and total sperm count. Masturbation was the most common behavior performed during pornography use in the present study and in other studies (40). However, a few studies have investigated the relationship between masturbation frequency during pornography use and semen parameters. A large online study on male sexual health in three European countries showed that frequent pornography use significantly increased the masturbation frequency among coupled men with decreased sexual desire (41). Additionally, higher masturbation frequency might increase the threshold of sexual arousal. Thus, we hypothesize that frequent masturbation might lead to frequent arousal and ejaculation, leading to continual hyperemia in the genitals and gonads, which could eventually affect spermatogenesis. Besides, frequent masturbation is usually accompanied with shorter abstinence time, which is also a risk factor for lower sperm concentration (20).

The present study showed that the frequency of masturbation when using pornography was negatively associated with sperm concentration while positively associated with morphological normal spermatozoa. It seems that this behavior has brought controversial effect to semen quality. Actually, it just reflects the physiology of sperm production. As the abstinence period grows, the accumulation of sperms leads to an increase in sperm concentration, while the accumulated sperms become senescent and lead to a decrease in the proportion of morphological normal spermatozoa. Vice versa, the masturbation behavior during using pornography decreased the abstinence period of the subjects, and consequently decreased the sperm concentration and increased the proportion of morphological normal spermatozoa.

The main strengths of this study were as follows. First, this was a follow-up study of MARHCS cohort. All of the participants were familiar with the study design, and they were well educated and could fully understand all of the questions, which make our results more credible. Second, our sample constituted a large healthy population with a narrow age range, and the selection of the same sampling season significantly reduced potential confounders. Third, the semen quality evaluation methods were used as per the WHO recommendations, which make our results more comparable with those of other studies.

There are some limitations to our study. First, the pornography use data were obtained *via* self-review, which raises the possibility of information bias. Secondly, the subjects of the present study were all recruited from the universities which were located in the university town of Chongqing, although the students came from different provinces all over China. The sampling season was restricted in summer and was several years ago. The population was highly homogenous, with similar ages, lifestyles, and education levels, which make it challenging to compare with other populations. Thirdly, each subject only offered one semen sample in each follow-up investigation, which might have introduced intra-individual bias to this study. Fourthly, sex is a sensitive topic for Chinese students to talk about. Thus, the pornography use data the students reported may be conservative, and the potential effects may have been underestimated. Although we adjusted for potential confounders on semen parameters and sex hormones, some other potential confounders might still exist, such as environmental exposure, psychological distress, and socioeconomic status. Whether the findings of the present study could be replicated in independent populations with different characteristics and circumstances await further studies. The results of the current study should be interpreted with caution, and further studies evaluating the relationship between pornography use and reproductive phenotypes should be conducted.

In conclusion, the present study showed that pornography use was quite common among college students in China. Our results showed pornography use was correlated with masturbation behavior and may lead to addictive behavior. This is also the first study to investigate the associations between pornography use and semen parameters. The results showed that pornography use was significantly associated with sex hormones in serum and semen parameters, which indicated that early and frequent pornography exposure may lead to adverse male reproductive outcome. Our results may have important implications for public health.

## Data Availability Statement

The raw data supporting the conclusions of this article will be made available by the authors, without undue reservation.

## Ethics Statement

The studies involving human participants were reviewed and approved by The Ethics Council of the Army Medical University. The patients/participants provided their written informed consent to participate in this study.

## Author Contributions

ZC and MM contributed to interpretation of the data and drafted the paper. The study was conceived and designed by ZC and JC. MM and QC contributed to statistical analyses. Data acquisition was conducted by ZC, MM, QC, XW, HY, NZ, LS, JL, and LA. All authors contributed to the article and approved the submitted version.

## Funding

This work was supported by the Fundamental Research Funds for the Central Universities, SWU [grant number 7110100301], National Natural Science Foundation of China [grant numbers 81502788], and National Key Research and Development program of China [grant number 2017YFC1002001].

## Conflict of Interest

The authors declare that the research was conducted in the absence of any commercial or financial relationships that could be construed as a potential conflict of interest.

## Publisher’s Note

All claims expressed in this article are solely those of the authors and do not necessarily represent those of their affiliated organizations, or those of the publisher, the editors and the reviewers. Any product that may be evaluated in this article, or claim that may be made by its manufacturer, is not guaranteed or endorsed by the publisher.
